# No midterm advantages in the middle term using small intestinal submucosa and human amniotic membrane in Achilles tendon transverse tenotomy

**DOI:** 10.1186/s13018-016-0463-1

**Published:** 2016-11-24

**Authors:** Yushu Liu, Yinbo Peng, Yong Fang, Min Yao, Robert W. Redmond, Tao Ni

**Affiliations:** 1 Department of Burns and Plastic Surgery, Shanghai Ninth People’s Hospital, Shanghai JiaoTong University School of Medicine, Mohe Road, No. 280, Baoshan District, Shanghai, 201900 People’s Republic of China; 2 Wellman Center for Photomedicine, Harvard Medical School, Massachusetts General Hospital, Boston, MA 02114 USA

**Keywords:** Small intestinal submucosa, Human amniotic membrane, Achilles tendon injury

## Abstract

**Background:**

The study was aimed to compare the effects of small intestinal submucosa (SIS) and human amniotic membrane (HAM) on Achilles tendon healing.

**Methods:**

A total of 48 New Zealand white rabbits were divided into two groups. A full-thickness transverse tenotomy was made at the right leg of the rabbits. Then, the laceration site was wrapped with HAM (P/A group) or SIS (P/S group). The ultimate stress (US) and Young’s modulus (*E*) of the tendons were detected for biomechanical analysis. Histological evaluation was performed using hematoxylin and eosin, immunohistochemical, and immunofluorescent stain. Expression of collagen I was detected by western blot analysis, and levels of inflammatory cytokines IL-1β, IL-6, and TNF-α were measured. Finally, adhesion formation was evaluated.

**Results:**

There were no significant differences in filamentous adhesion, cross-sectional areas of the laceration sites, levels of inflammatory response, and collagen type I expression between the P/A and P/S groups (*p* > 0.05). Compared with the P/A group, the US and *E* values were significantly higher in the P/S group at day 7 (*p* < 0.05) and at day 14 (*p* < 0.05). In addition, vascularity was significantly higher in the P/S group than that in the P/A group at day 3 (*p* < 0.05), day 7 (*p* < 0.01), and day 9 (*p* < 0.05).

**Conclusions:**

SIS showed superior biomechanical properties and neovascularization over HAM in treatment of Achilles tendon injury in the early stage of healing.

## Background

As a common injury, the incidence of Achilles tendon rupture has increased during the past few decades. Achilles tendon rupture which often locate on the left side occurs more frequently in men when they are 30 or 40 years old [1]. Most Achilles tendon ruptures happen during sports [2], and the incidence is increasing as a result of an increase of sport activities [3].

Controversial discussion on treating acute Achilles tendon ruptures between nonoperative or operative repairs still remains [4, 5]. Nonoperative treatment has been reported to be related to long periods of cast immobilization and high rates of rupture resulting in stiff ankles and weak calf muscles [5, 6]. Surgical repair could minimize the gap between the ends of the tendon, giving rise to intensive tendon healing and decreased scar formation [4, 5]. With these controversies, researchers suggest a tendency for operative treatment [7, 8].

Many materials including human amniotic membrane (HAM), small intestinal submucosa (SIS), polyhydroxyethyl methacrylate membranes, and polypropylene mesh have been used as tendon graft materials to improve healing of tendon defects [9]. As the innermost layer of the placenta, HAM has anti-inflammatory, anti-angiogenic, and anti-bacterial effects [10] and has been known to support cellular migration, adhesion, and proliferation for healing in tissue defect sites [11], which lead HAM to be used in a wide variety of clinical scenarios including tendon repair and peritendinous adhesion of flexor tendons impedance [12, 13]. Nowadays, SIS has become a popular biological dressing in the reconstruction field. Researches have demonstrated that SIS can successfully remodel into functional tendon [14] and SIS is also an ideal scaffold for tendon repair [15]. Thus, according to the previous experiment, HAM and SIS could be applied to the Achilles tendon repair, but which one has more advantages is not fully understood.

In the current study, we aimed to test either HAM or SIS is preferable for the tendon repair. Thus, we investigated the healing effect of HAM and SIS on the ruptured Achilles tendon in rabbit models through adhesion evaluation, biomechanical, histological, and histomorphometrical analysis.

## Methods

### Animals

A total of 48 New Zealand white rabbits weighing 2.0–2.5 kg were purchased from Song Lian experimental animal farm (Shanghai, China). These animals were fed with normal fodder and tap water in standard hutches. The experiment was conducted under the protocol approved by our university. All studies were performed in agreement with the Principles of Laboratory Animal Care and guidelines established by the Institutional Animal Care and Use Committee of the university.

### Preparation of biomaterials

Fresh HAM (purchased from Jiangxi Ruiji Biological Engineering Technology Co., Ltd.) was obtained from placentas in healthy donor mothers without infectious disease. Peeling the AM away from the chorion, the membrane was washed with sterile phosphate-buffered saline (PBS) to remove the blood clots and then permeated into 0.05 % trypsin + 0.02 % ethylenediaminetetraacetate (EDTA) at 37 °C for 2 h. After washing with sterile PBS containing streptomycin (0.1 mg/ml) and amphotericin B (0.25 μg/ml) for three times, the sterilized nitrocellulose filter paper with adherent HAM was stored in 50 % Dulbecco’s modified Eagle medium (DMEM) and 50 % glycerol at −80 °C.

The porcine small intestine was obtained and harvested from healthy pigs at the age of 3–6 months within 4 h of sacrifice. SIS was obtained using a longitudinal wiping motion with a scalpel handle and moistened gauze after removing the tunica serosa and muscularis. Submucous membrane was put into a solution containing methanol and chloroform (1:1, *V*/*V*) for 12 h and washed with ddH_2_O. After that, the membrane was incubated in 0.05 % trypsin and 0.05 % EDTA at 37 °C for 12 h and further treated with 0.5 % SDS in 0.9 % sodium chloride by shaking for 4 h. After removal of the detergent, the membrane was permeated through 0.1 % peroxyacetic acid and 20 % ethanol for 30 min and rinsed with saline solution.

### Surgical procedure

Anesthesia was induced with an intravenous injection of 3 % pentobarbital sodium (1 ml/kg). After the right hind leg was shaved under sterile conditions, a 5-cm linear skin incision was created and a full-thickness transverse tenotomy was made at the mid-tendon point. Rabbits were divided into two groups in average (*n* = 24 in each group). Group P/A: a 2 cm × 2 cm section of the HAM soaked into 0.1 % rose bengal (RB) for 1 min. A single 5–0 Vicryl (Ethicon, USA) suture was placed to bring the tendon stumps into contact, and the HAM was permeated with 0.1 % RB wrapped around the tendon and bonded to the tendon surface. The group P/S was similar to the group P/A except that HAM was replaced with SIS. After a surgical wound is closed by 4–0 silk suture, the experimental leg was immobilized via plaster of paris bandage to make sure the hip joint is at 90° flexion and the ankle is at 180° straight. After a month, the plaster of paris bandage was removed, and animals were allowed to be active. At 7, 14, 28, and 56 days postoperation, six rabbits were respectively sacrificed. The experimental leg was opened again. After the repaired region is photographed, the Achilles tendon was harvested with part of the gastrocnemius muscle and calcaneus insertion. Then, the tendon samples were tested for mechanical properties. Finally, the sample was divided into two parts, one was immobilized by 4 % paraformaldehyde and another was frozen and stored at −80 °C.

### Biomechanical analysis

The Achilles tendon came from gastrocnemius in the calcaneal stop and wrapped around a single layer of gauze at both ends to prevent glide of the Achilles tendon in the process of tension detection. After the width and thickness of the tendon were measured using a digital caliper, the tension of each repaired tendon was also tested by an Instron 4301 machine. The apparatus has a maximum load of 500 N and a minimum resolution of 0.01 N. The force-strain curve was collected for each sample by a connected computer with a speed of 10 mm/min. The apparatus quitted automatically when reached the maximum tension of each sample, and the vertex of the curve was regarded as the ultimate stress (US). The Young’s modulus (*E*), represented the slope coefficient of the stress-strain curve, is calculated by the equation: *E* = (FL)/(*S*△*L*), where *F* is the ultimate stress, *L* is the original in sample length, △*L* is the change length of the sample, and *S* is the cross-sectional area of the sample which is acquired by multiplied width with thickness.

### Histology, histomorphometry, and immunofluorescent analysis

Samples were firstly immobilized by 4 % paraformaldehyde for 48 h at room temperature, then dehydrated in ethanol, and embedded in paraffin. Sagittal sections about 4 μm were cut for hematoxylin and eosin (HE) and immunohistochemistry at days 7, 14, 28, and 56 after operation and immunofluorescent staining analysis at postoperative days 3, 7, and 9. For HE, samples were stained with HE. For immunohistochemistry, tissue was fixed in paraformaldehyde and an enzymatic antigen retrieval step was performed. Samples were then blocked using 5 % bovine serum albumin (BSA) for 1 h at room temperature and incubated with collagen I antibody (1:200, Cambridge, MA, US) at 4 °C overnight. For immunofluorescent staining, vascularity was quantified by a morphometric stereological analysis on paraffin sections stained with anti-CD31 monoclonal antibody (1:50). A secondary antibody conjugated with fluorescein isothiocyanate (FITC) was applied at room temperature for 45 min.

### Western blot analysis

The quantity of collagen I in each samples of repaired region was analyzed by western blot. Sample proteins were transferred to a polyvinylidene fluoride (PVDF) membrane for 70 min after being electrophoresed through a 6 % sodium dodecyl sulfate (SDS)-polyacrylamide gel electrophoresis (PAGE) gel. Then, the membranes were incubated with collagen I antibody (1: 200) at 4 °C overnight after being blocked by 5 % milk for 1 h at room temperature. GAPDH was regarded as an internal reference.

### Enzyme-linked immunosorbent assay (ELISA) analysis

The protein levels of IL-1β, IL-6, and TNF-α in the repair site were measured by ELISA, following the manufacturer’s instructions (USCN, Wuhan, China).

### Adhesion evaluation

The adhesion grading system was as follows: grade 0, no adhesion; grade 1, filmy, transparent, and avascular; grade 2, opaque, translucent, and avascular; grade 3, opaque and capillaries present; grade 4, opaque and larger vessels present. The evaluation of scope adhesion formation was independently observed by two surgeons blinded to the details of the study when animals were sacrificed.

### Statistical analysis

Statistical analysis of significant difference between groups was determined using independent sample *t* test that was used with SPSS 16.0 with significance set at a level of *p* < 0.05.

## Results

### Macroscopic findings of tendon repair

**Fig. 1:**
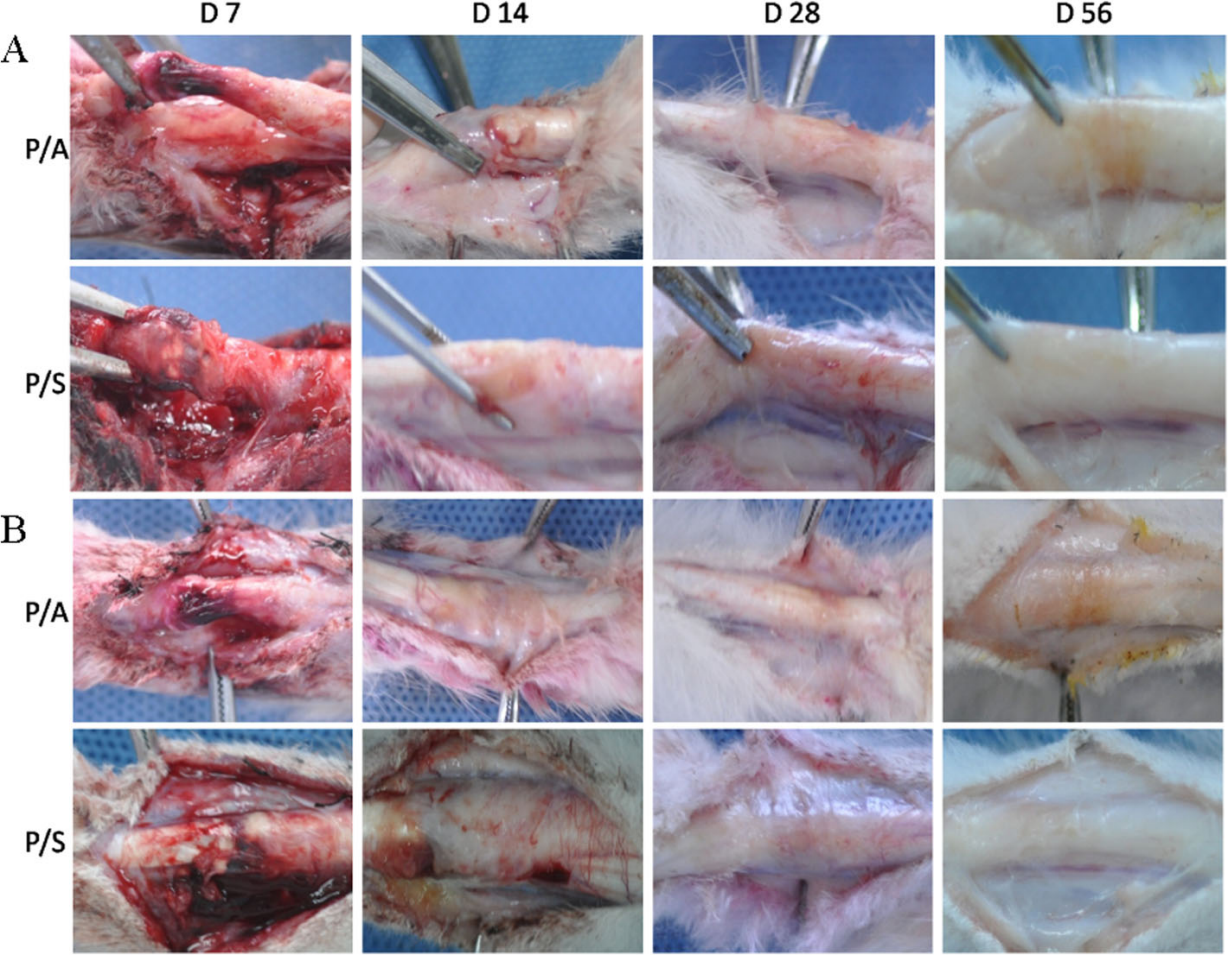
Macroscopic findings of tendon repair from the frontal (**a**) and lateral (**b**) view of human amniotic membrane (HAM) and small intestinal submucosa (SIS) at different groups

From the frontal view of tendon repair, no dehiscence or sign of infection was observed in any group at the observed time postoperatively (Fig. 1a). The remaining blood was still observed at 7 days after the operation in all groups. During the 14 days postoperation, HAM or SIS was difficult to distinguish as the surgical areas were opaque. The repair site was mostly healed at 28 days and the slender tendons were found in the groups P/A and P/S at 56 days. The lateral view (Fig. 1b) was used to evaluate the adhesion formation. Filamentous adhesion which was easy to separate was observed in P/A or P/S at 28 and 56 days after operation, respectively. As a month of activity without external fixation, the tendon repair site showed less adhesion formation at 56 days than that in 28 days.

### Postoperative adhesion score and biomechanical properties

**Fig. 2:**
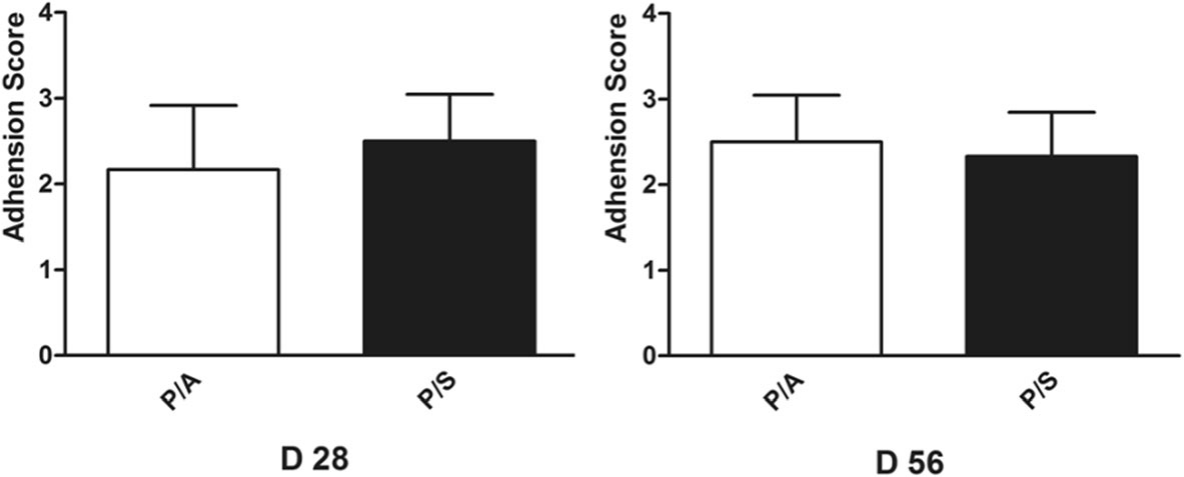
Post-surgical adhesion score at 28 and 56 days

Post-surgical adhesion score showed that there was no difference of adhesion scores at day 28 (*p* > 0.05) or day 56 (*p* > 0.05) between the P/A and P/S groups (Fig. 2).

**Fig. 3:**
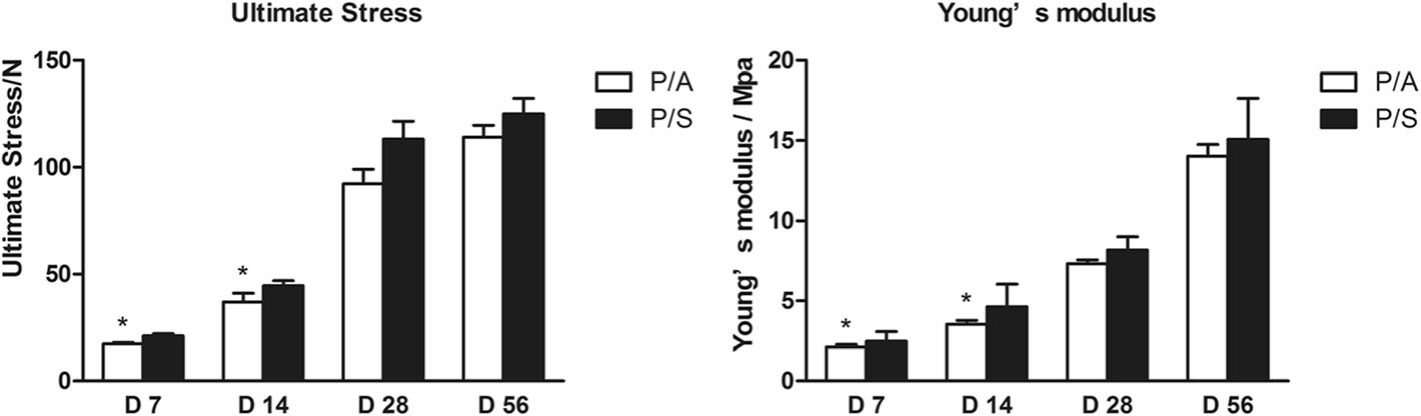
The ultimate stress and Young’s modulus of the tendons in the P/A and P/S groups at days 7, 14, 28, and 56. *p < 0.05

The biomechanical property of the tendons presented by US and *E* were shown in Fig. 3. Compared with the P/A group, the US and *E* values were significantly higher in the P/S group at day 7 (*p* < 0.05) and at day 14 (*p* < 0.05). There was no significant difference of US or *E* between the two groups at day 28 and day 56 (*p* > 0.05).

### Inflammatory response

**Fig. 4:**
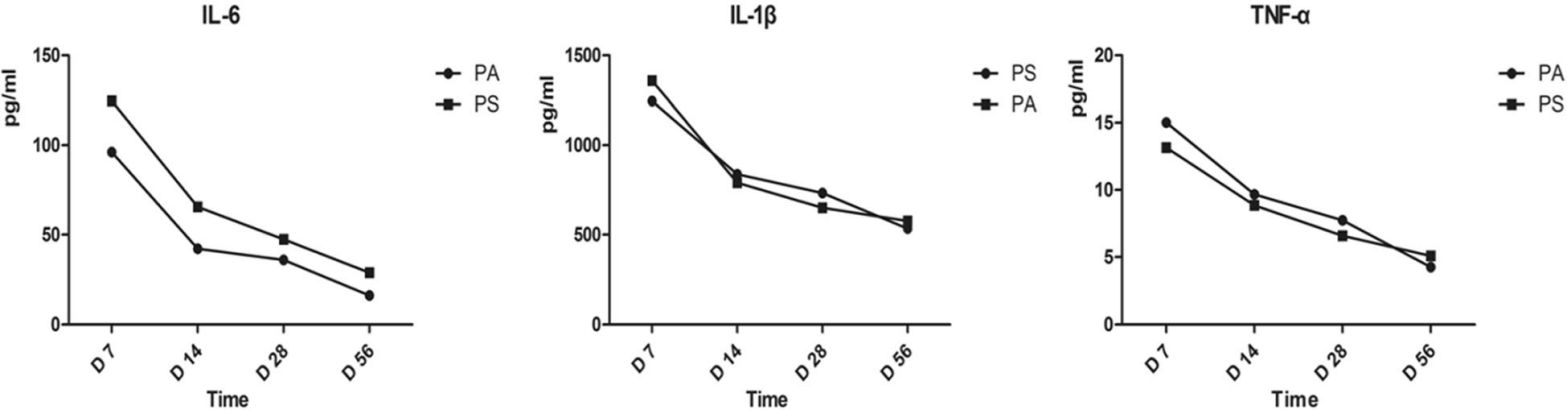
The changes of IL-1β, IL-6, and TNF-α level in the P/A and P/S groups at days 7, 14, 28, and 56 after operation

To study the variation of inflammatory response after operation, we tested the levels of inflammatory cytokines IL-1β, IL-6, and TNF-α. Each cytokine showed consecutive decline trend as the extension of time after surgery in all groups (Fig. 4). A rapid downward trend was found in the P/A group and P/S group for any cytokines from 7 to 14 days postoperation. At day 56, there were no significant differences of the levels of IL-1β, IL-6 and TNF-α between the P/A and P/S groups (*p* > 0.05).

### Histology and histomorphometry

**Fig. 5:**
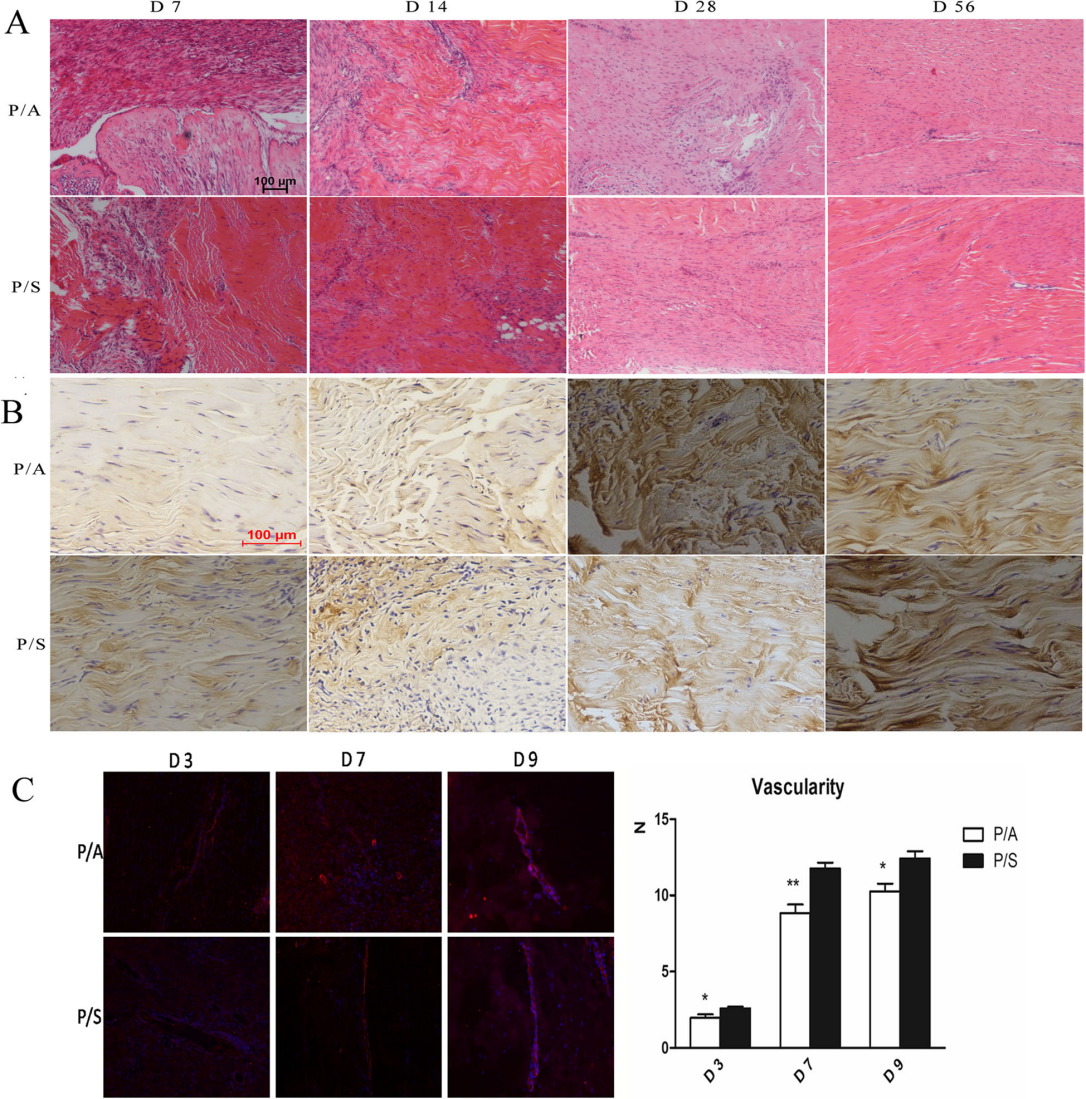
Histological sections with hematoxylin and eosin staining (**a**) and immunohistochemical staining (**b**) for collagen type I at 7, 14, 28, and 56 days. *Scale bar*, 100 μm. **c** The immunofluorescent stain of CD31 at postoperative 3, 7, and 9 days in two groups. *p < 0.05, **p < 0.01

The process of tendon repair was revealed by HE and immunohistochemical staining in Fig. 5a, b, respectively. The HE stain revealed that tendon stumps sieged by inflammatory cells could be clearly found in both groups at day 7. At day 14, stumps migrated to the repair site but it still not completely healed. The palingenetic tendon tissue is arranged in a random orientation. Diffuse inflammation cells were decreased evidently and the tendon stumps tended to heal at day 28. The arrangement of fibroblasts and collagen fibers were more organized in the P/A and P/S groups at day 56.

In the immunohistochemical staining (Fig. 5b), a small amount of collagen I was found in the two groups at day 7. At the 14 days after surgery, the expression of collagen I was increased, and it was mainly distributed at the end of edge in the P/S group, and in the P/A group, the distribution was scattered. At day 28, the collagen I was in a patchy distribution, and the wound was scattered. At day 56, the arrangement of collagen I in the two groups was wave-like.

The immunofluorescent stain of CD31 (Fig. 5c) showed that vascularity was found in the two groups at days 3, 7, and 9. In addition, vascularity was significantly higher in the P/S group than that in the P/A group at days 3 (*p* < 0.05), 7 (*p* < 0.01), and 9 (*p* < 0.05).

### Expression of collagen I

**Fig. 6:**
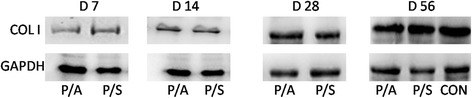
Western blotting assay for expression of collagen I at 7, 14, 28, and 56 days in the P/A and P/S groups

Western blotting assay showed that at day 7, the P/S group showed a higher level of collagen I compared with the P/A group (Fig. 6). However, no significant difference was seen between the P/A group and P/S group at days 14, 28, and 56, which meant that photochemical tissue bonding (PTB) combined with biological dressing did not promote the collagen synthesis of tendon healing.

## Discussion

In the present study, we investigated the healing effect of HAM and SIS on the ruptured Achilles tendon in rabbit models. We found that the biomechanical property of the tendons was significant higher in the P/S group at postoperative day 7 and day 14 than the P/A group. In addition, vascularity was significantly higher in the P/S group than that in the P/A group at days 3, 7, and 9. The results of the present study indicate that SIS applied to the laceracted tendons as a biologic scaffold has better treatment effect at Achilles tendon rupture than HAM in a rabbit model.

As a naturally occurring extracellular matrix, the porcine SIS plays an important role as a scaffold next to which the recipient site is acceptable to rebuild functionally and structurally suitable tissue [16]. The SIS device is composed of collagen (about 90 %), glycosaminoglycans, fibronectin, and growth factors including transforming growth factor β (TGF-β), vascular endothelium growth factor, and fibroblast growth factor 2. SIS has been used in various different organ system repairs [17]. Previous research has revealed promising findings in orthopedic soft tissue applications using SIS for Achilles tendon defect repair in dogs [18] and for repair of a musculotendinous tissue in a canine Achilles tendon repair model [15] . In this study, the SIS device was implanted as a resorbable scaffold and acted as a regenerative response, leading to collagen I-rich, connective tissue histologically similar with induced using HAM repair.

Biomechanical testing demonstrated that the US and *E* values were significant higher in the P/S group at postoperative day 7 and at day 14 than those in the P/A group. Previous investigations applying HAM in tendon healing in a rabbit Achilles tendon model found that the biomechanical modulus of the HAM treatment group was higher at 4 weeks than that of the control group [12], showing the treatment effect of HAM. In addition, Zalavras et al. [14] revealed the progressive remodeling of the SIS-regenerated tendon in a rat model. In this research, we found no difference between the P/A and P/S groups in adhesion formation. Previous study has found that HAM could induce down-regulation of TGF-β signaling, which contributes to wound healing, such as the stimulation of collagen production and recruitment of fibroblasts [19]. On the other hand, TGF-β has been found prospective to decrease the formation of peritendinous adhesions on the flexor tendons after surgery [20]. It may hint that the effect of HAM and SIS on TGF-β may lead to less adhesion formation. Another important result is the higher strength with SIS after operation day 7 and day 14 biomechanical studies than the P/A group. These findings indicate that application of SIS is superior to HAM in tendon repairs.

The healthy Achilles tendon tissue is short of blood supply, and its nutrition mainly comes from the synovial membrane dispersion and blood vessels dispersion. When the Achilles tendon ruptures, the blood supply is interrupted and the blood vessel is damaged. New vascular network and granulation tissue formed by neovascularization provide the necessary nutrients for tendon repair. Generally, neovascularization occurs in the early stage of Achilles tendon healing, and the monitoring of new blood vessels can reflect the speed of Achilles tendon healing and indirectly reflect the impact of biological materials on the healing of Achilles tendon. In our present study, neovascularization in the P/S group was significantly higher compared with the P/A group. Previous investigator has revealed that transplantation of SIS is an effective bioscaffold as a body wall repair device [21]. The SIS-regenerated tendons in our rabbit model showed fibroblastic ingrowth, collagen I deposition, reduction of inflammatory cytokines (IL-1β, IL-6 and TNF-α) levels, and neovascularization. It has been verified that fibroblastic cells appearing at the healing position are stemmed from undifferentiated mesenchymal cells which were derived from the recipient site [22]. Thus, findings of the current study are consistent with the hypothesis of other studies that porcine xenograft acts as an agent with regenerative potential which plays an important role in host tissue ingrowth [16, 23]. However, our study is observational, and more researches with set criteria quantified by blinded observers are still needed to be clarified.

There are still some limitations in the current study. First, the number of animals at each timepoint is small and quantitative evaluation of reparative cells and collagen proliferation using objective analyses are lacking. Second, the model in this study is not exactly clinically relevant to Achilles tendon ruptures in humans, and the Achilles tendon does not rupture in a clean fashion. Third, no verification in clinical human practice is one limitation of this study, and thus the long-term effects of SIS on tendon-healing are needed to be investigated in the future clinical practice. In addition, the addition of more bulk to the repair can, in humans, result in more difficult closure of the wound and give rise to a greater rate of infection.

## Conclusions

In conclusion, SIS was effective as an agent for the treatment of Achilles tendon injury in a rabbit model. In addition, SIS showed superior biomechanical properties and neovascularization over HAM in treatment of Achilles tendon injury in the early stage of healing. However, further animal and clinical studies are still needed to explore the role of SIS as a therapeutic option for the treatment of Achilles tendon injury.

## Abbreviations

BSA: Bovine serum albumin
FITC: Fluorescein isothiocyanate
HAM: Human amniotic membrane
PVDF: Polyvinylidene fluoride
SIS: Small intestinal submucosa
